# Profiling the composition of resistome and bacteriome in the upper respiratory tract of domestic cats with respiratory signs in China

**DOI:** 10.20517/mrr.2025.04

**Published:** 2025-07-28

**Authors:** Qiuyan Li, Dengyuan Zhou, Longlong Cao, Yongfan Li, Jiakang Li, Jing Ye, Huanchun Chen, Jiangchao Zhao, Shengbo Cao, Zhong Peng

**Affiliations:** ^1^National Key Laboratory of Agricultural Microbiology, College of Veterinary Medicine, Huazhong Agricultural University, Wuhan 430070, Hubei, China.; ^2^Hubei Hongshan Laboratory, Wuhan 430070, Hubei, China.; ^3^Frontiers Science Center for Animal Breeding and Sustainable Production, The Cooperative Innovation Center for Sustainable Pig Production, Huazhong Agricultural University, Wuhan 430070, Hubei, China.; ^4^Department of Animal Science, Division of Agriculture, University of Arkansas, Fayetteville, AR 72701, USA.; ^#^Authors contributed equally.

**Keywords:** Domestic cats, genomic surveillance, resistome, bacteriome, China

## Abstract

**Aim:** Domestic cats, among the most popular pets globally, may harbor antimicrobial resistance genes (ARGs) and zoonotic pathogens that impact human health. This study aims to investigate the resistome and bacteriome composition in the upper respiratory tract of domestic cats with respiratory signs in China.

**Methods:** We performed metagenomic sequencing on 1,454 oropharyngeal-nasal swabs from cats with respiratory signs across diverse living conditions in 22 Chinese provinces. Resistome and bacteriome profiles were analyzed using these sequencing data.

**Results:** We characterized the resistome and bacteriome in the upper respiratory tract of cats, identifying a wide range of ARGs - including those conferring resistance to last-resort antibiotics {e.g., carbapenems (*bla*_NDM_, *bla*_OXA-244_, *bla*_VIM-13_, *bla*_VIM-33_), colistin (*mcr*), and high-level tigecycline [MIC ≥ 4 µg/mL; *tet*(X3), *tet*(X4), *tet*(X5), *tet*(X6)]}. Additionally, we detected numerous bacterial species of public health concerns, including the six leading antimicrobial resistance-associated pathogens (*Escherichia coli*, *Staphylococcus aureus*, *Klebsiella pneumoniae*, *Streptococcus pneumoniae*, *Acinetobacter baumannii*, *Pseudomonas aeruginosa*) and other high-burden pathogens linked to global morbidity, mortality, and therapeutic challenges.

**Conclusion:** The findings highlight the potential zoonotic risks posed by cats. Including monitoring of this companion species within the One Health approach to address public health concerns is necessary.

## INTRODUCTION

Antimicrobial resistance (AMR) is one of the most critical global public health threats^[1]^. Without urgent intervention, drug-resistant infections are projected to cause 10 million deaths annually worldwide by 2050, with a cumulative economic loss of 100 trillion USD^[2]^. A recent systematic analysis revealed that lower respiratory infections were the leading AMR-associated infectious syndrome in 2019, responsible for over 1.5 million deaths^[3]^. The same study identified *Escherichia coli*, *Staphylococcus aureus*, *Klebsiella pneumoniae*, *Streptococcus pneumoniae*, *Acinetobacter baumannii*, and *Pseudomonas aeruginosa* as the six deadliest AMR-related pathogens^[3]^.

Domesticated animals, particularly pets, are recognized as potential reservoirs for transmitting antimicrobial-resistant pathogens to humans^[4,5]^. Among them, domestic cats (*Felis catus*), one of the world’s most popular household pets, have experienced a dramatic population surge over the past 40 years. By 2021, the global cat population reached 600 million, with 400 million in Asia^[6]^. In China, the number of household cats exceeded 96 million in 2021 and continues to rise^[7]^, alongside a substantial stray cat population. Given their close interaction with humans, cats harbor antimicrobial resistance genes (ARGs) and zoonotic pathogens that pose public health risks. For example, studies have documented that companion cats can carry human-associated pathogens and multidrug-resistant bacteria, including MRSA and β-lactam-resistant *Enterobacteriaceae*^[8,9]^. Recent evidence also highlights the transmission of resistomes from cats to their owners and living environment^[10]^. While the oral microbiota of healthy cats is predominantly composed of *Pasteurellaceae*, *Moraxellaceae*, *Thermomonas*, and *Comamonadaceae*^[11]^, an increasing number of human infections - particularly following cat bites - have been linked to bacterial species within these families^[12,13]^. Despite these risks, comprehensive surveillance of AMR in domestic cats remains limited.

Recently, metagenomic sequencing, combined with bioinformatic analysis, has emerged as a powerful tool for AMR and microorganism surveillance^[14,15]^. In this study, we employed nationwide metagenomic sequencing to profile the resistome and bacteriome in the upper respiratory tract of domestic cats across China. Our findings aim to elucidate the public health risks posed by these ubiquitous companion animals.

## METHODS

### Ethics statement

This study was approved by the Animal Management and Ethics Committee of Huazhong Agricultural University (Approval ID: HZAUCA-2023-0005).

### Sample collection, treatment, and metagenomic sequencing

**Figure 1:**
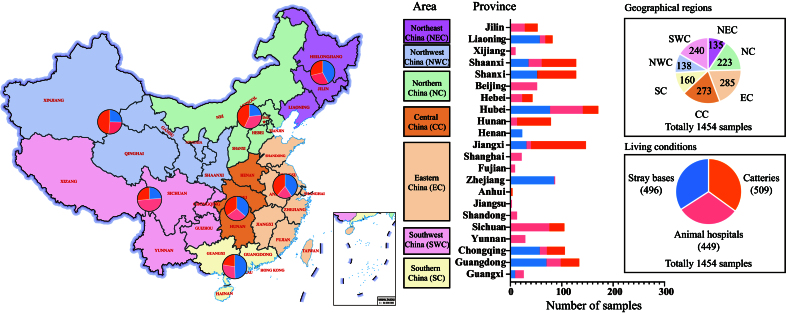
Distribution of oral-nasal swabs collected from domestic cats in various living conditions across different regions in China. Different Chinese regions: NEC - Northeast China, NC - Northern China, EC - Eastern China, CC - Central China, SC - Southern China, NWC - Northwest China, SWC - Southwest China.

From September 1 to October 30, 2022, we collected 1,454 oropharyngeal-nasal swabs from domestic cats exhibiting respiratory signs across diverse environments in 22 Chinese provinces [Figure 1 and Supplementary Table 1]. Sampling sites include: catteries (509 samples), animal hospitals (449 samples), and stray bases (496 samples). Samples were geographically distributed as follows: Northeast China, 135 samples (39 samples from catteries, 39 samples from animal hospitals, 57 samples from stray bases); Northern China, 223 samples (96 from catteries, 75 from animal hospitals, 52 from stray bases); Eastern China, 285 samples (111 from catteries, 58 from animal hospitals, 116 from stray bases); Central China, 273 samples (96 from catteries, 77 from animal hospitals, 100 from stray bases); Southern China, 160 samples (36 from catteries, 45 from animal hospitals, 79 from stray bases); Northwest China, 138 samples (67 from catteries, 36 from animal hospitals, 35 from stray bases); Southwest China, 240 samples (64 from catteries, 119 from animal hospitals, 57 from stray bases). Within each geographical region, samples from catteries, animal hospitals, and stray bases were pooled separately, yielding 63 pools for analysis [Figure 1].

DNA extraction was performed using the cetyltrimethylammonium bromide protocol^[16]^. DNA quality/quantity was assessed through 1% agarose gel electrophoresis, NanoDrop2000 (Thermo Fisher Scientific, Waltham, US), and Agilent 2100 Bioanalyzer (Agilent, Santa Clara, US), respectively. Libraries were prepared with the NEBNext® Ultra^TM^ DNA Library Prep Kit (NEB, Ipswich, US; 350 bp insert size) and sequenced on an Illumina Nova600 PE150 platform (Illumina, San Diego, China) employing the paired-end 150 bp strategy. All data were deposited into the NCBI Sequence Read Archive (SRA) database (Bioproject: PRJNA998709; Accessions: SRR33674915-SRR33674924, SRR33696099-SRR33696115, SRR34261881-SRR34261890).

### Sequence assembly and identification of ARGs

Metagenomic sequencing generated ~10.84 gigabytes of (Gb) reads per sample [Supplementary Table 2]. The quality of the raw reads was assessed using FastQC (v 0.12.1) (https://github.com/s-andrews/FastQC), and reads containing low-quality and adaptors were eliminated using Trimmomatic (quality score ≥ 20)^[17]^. Clean reads were then assembled using MEGAHIT (v1.1.2; parameters: -k-min 61 -min- contig-len 1000)^[18]^, with assembly quality evaluated via QUAST 5.2.0^[19]^. Contigs carrying ARGs were identified following a protocol outlined in a previously published article^[20]^. Briefly, contigs derived from sequence assembly were utilized to predict open reading frames (ORFs) using Prodigal^[21]^. ARG-like ORFs were identified by comparing the amino acid sequences of the predicted ORFs against the Structured ARG (SARG) database (v 2.0)^[22]^ using Diamond v0.8.35^[23]^, with the following BLASTP parameters: e-value ≤ 10-10, an identity ≥ 80%, and query coverage ≥ 70%^[20]^.

### Identification of bacterial species

For bacterial species characterization, predicted ORFs were matched against the NCBI NR database using Diamond (version 0.8.35; E-value ≤ 1e-5)^[23]^. The alignment outcomes were subsequently assessed utilizing the Lowest Common Ancestor (LCA) algorithm implemented in MEGAN^[24]^. Taxonomic lineage details were retrieved for the top BLAST hit of each contig. DNA abundances were depicted visually using Krona^[25]^. The bacterial taxonomic contributors to the ARGs were identified employing FishTaco^[26]^ with default parameters.

### Statistical analysis

Transcripts per million (TPM) per predicted gene was calculated as a proxy for gene abundance. TPM was defined as the number of metagenomic reads mapped to a given gene (× 10^6^), divided by the gene’s length, and normalized by the sum of all reads mapped to all genes, also adjusted by their respective lengths, as previously described^[27,28]^. Analysis of similarities (ANOSIM) was used to evaluate whether the grouping makes sense (0 < *R* < 1)^[29]^. For comparing the abundances between two groups, Mann-Whitney U test (known as the Wilcoxon rank-sum test) was performed using pairwise multiple comparison adjustments according to the Benjamini-Hochberg procedure, as previously described^[30,31]^. *P* < 0.05 (*) indicates statistically significant.

## RESULTS

### Composition of resistome in the upper respiratory tract of cats in China

**Figure 2:**
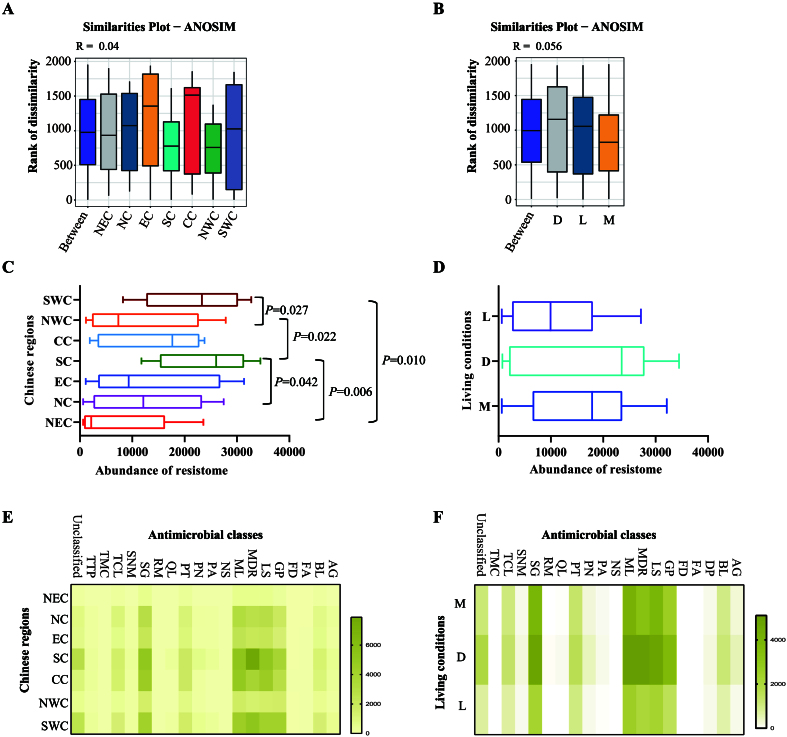
Composition of the resistome in the upper respiratory tract of domestic cats determined by metagenomic sequencing. (A and B) ANOSIM evaluating the resistomes in the upper respiratory tract of domestic cats from different Chinese regions (A) and/or different living conditions (B); (C and D) Abundances of the resistomes in the upper respiratory tract of domestic cats from different Chinese regions (C) and/or different living conditions (D); (E and F) Heatmaps showing the abundances of genes conferring resistance to different antimicrobial classes in the upper respiratory tract of domestic cats from different Chinese regions (E) and/or different living conditions (F). Mann-Whitney U test was performed to compare the abundances between two groups, using pairwise multiple comparison adjustments according to the Benjamini-Hochberg procedure. The significance level was set at a *P* value of < 0.05 (*) and *P* > 0.05 is not marked. Different Chinese regions: NEC - Northeast China, NC - Northern China, EC - Eastern China, CC - Central China, SC - Southern China, NWC - Northwest China, SWC - Southwest China. Different living conditions: M - catteries, D - animal hospitals, L - stray bases. Different antimicrobial classes: AG - aminoglycosides, BL - β-lactams, FA - fusaric acids, FD - fusidanes, GP - glycopeptides, LS - lincosamides, MDR - multidrug resistant phenotype, ML - macrolides, NS - nucleosides, PA - phosphonic acids, PN - phenicols, PT - peptides, QL - quinolones, RM - rifamycins, SGS - streptogramins, SNM - sulfonamides, TCL - tetracycline, TMC - tetracenomycin, TTP - trimethoprim. ANOSIM: Analysis of similarities.

Following assembly, an average of 107,726 contigs with an average length of 136,712,231 bp (average *N*_50_: 2,085 bp) were generated for each pool [Supplementary Figure 1 and Supplementary Table 2]. ARG-like ORFs identified from the assembled contigs showed an average sequence identity of 93.39% (ranged: 80.00%-100%) and an average coverage of 98.35% (ranged: 70.24%-100%) [Supplementary Table 3]. ANOSIM analysis confirmed the validity of sample grouping by both geographic regions (*R* = 0.04) and living conditions (*R* = 0.056) [Figure 2A and B]. Subsequent comparison of resistome abundances using the Mann-Whitney U test revealed significant regional variations [Figure 2C]. Specifically, cats from Northeast China showed lower resistome abundances compared to those from Southern and Southwest China, while Southern China cats exhibited higher resistome levels than those from Northwest and Northern China (*P* < 0.05). No significant differences in resistome abundance were detected among different living conditions [Figure 2D].

After removing the duplicate genes, we identified 444 unique ARGs conferring resistance to 16 antimicrobial classes: aminoglycosides, β-lactams, diaminopyrimidines, glycopeptides, lincosamides, macrolides, nucleosides, peptides, phenicols, phosphonic acids, quinolones, rifamycin, streptogramins, sulfonamides, tetracenomycins, and tetracyclines [Supplementary Figure 2]. Notably prevalent were ARGs conferring multidrug resistance [Figure 2E]. High detection rates were also observed for resistance genes targeting aminoglycosides, β-lactams, glycopeptides, macrolides, lincosamides, peptides, phenicols, streptogramins, and tetracyclines [Figure 2E]. Regional variations in ARG prevalence across antimicrobial classes were evident [Figure 2E], while differences in the distribution of multidrug resistance genes were observed among cats from different living conditions [Figure 2F].

### Distribution of public health concern ARGs in the upper respiratory tract of cats

**Figure 3:**
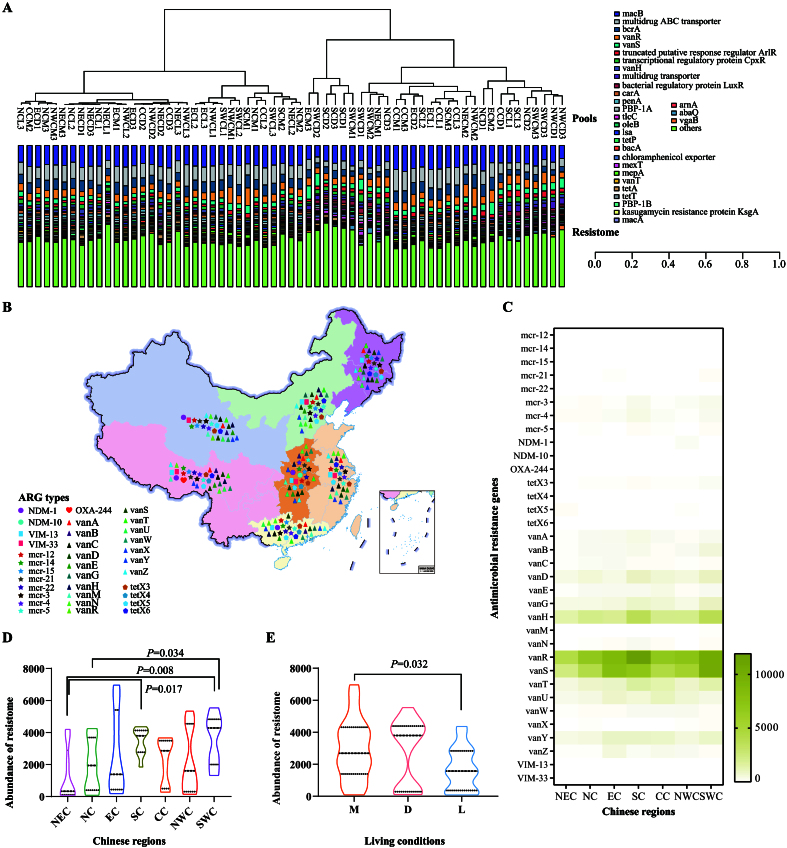
Detection of ARGs in the upper respiratory tract of cats in various Chinese regions. (A) A bar graph displaying the distribution of identified ARGs in the upper respiratory tract of cats across different Chinese regions (Only those with abundances ranking among the top thirty are shown); (B) A geographic map illustrating the detection of ARGs conferring resistance to last-resort antimicrobials (carbapenems, colistin, high-level tigecycline, vancomycin) in samples collected from cats in diverse living conditions in China; (C) A heatmap presenting the abundances of ARGs conferring resistance to last-resort antimicrobials (carbapenems, colistin, high-level tigecycline, vancomycin) in the upper respiratory tract of domestic cats from various Chinese regions; (D) A violin plot demonstrating the abundances of ARGs conferring resistance to last-resort antimicrobials (carbapenems, colistin, high-level tigecycline, vancomycin) in the upper respiratory tract of domestic cats across different Chinese regions; (E) A violin plot showing the abundances of ARGs conferring resistance to last-resort antimicrobials (carbapenems, colistin, high-level tigecycline, vancomycin) in the upper respiratory tract of domestic cats in different living conditions. Mann-Whitney U test was performed to compare the abundances between two groups, using pairwise multiple comparison adjustments according to the Benjamini-Hochberg procedure. The significance level was set at a *P* value of < 0.05 (*) and *P* > 0.05 is not marked. Different Chinese regions: NEC - Northeast China, NC - Northern China, EC - Eastern China, CC - Central China, SC - Southern China, NWC - Northwest China, SWC - Southwest China. Different living conditions: M - catteries, D - animal hospitals, L - stray bases. ARGs: Antimicrobial resistance genes.

We also identified several ARGs conferring resistance to critically important agents listed by the World Health Organization (WHO) as the highest priority for human medicine^[32]^. These included genes responsible for resistance to 3rd/4th/5th-generation cephalosporins (e.g., *bla*_CTX-M_, *bla*_TEM-1_, *bla*_SHV-1_, *bla*_VEB-5_, *bla*_GES-13_, *bla*_OXA-9/59/209_), vancomycin (e.g., *vanA*, *vanB*, *vanC*, *vanD*, *vanE*, *vanG*, *vanH*, *vanM*, *van*N, *vanR*, *vanS*, *vanT*, *vanU*, *vanV*, *vanX*, *vanY*, *vanZ*), macrolides and ketolides (e.g., *ermA*, *ermB*, *ermF*), polymyxins (e.g., *mcr-1.2*, *mcr-1.4*, *mcr-1.5*, *mcr-2.1*, *mcr-2.2*, *mcr-3*, *mcr-4*, *mcr-5*), and quinolones (e.g., *qnrA*, *qnrB*, *qnrS*) [Figure 3A and Supplementary Figure 2]. Of particular concern were ARGs conferring resistance to the last-resort antimicrobials, including carbapenems (*bla*_NDM-1_, *bla*_NDM-10_, *bla*_OXA-244_, *bla*_VIM-13_, *bla*_VIM-33_), colistin (*mcr-1.2*, *mcr-1.4*, *mcr-1.5*, *mcr-2.1*, *mcr-2.2*, *mcr-3*, *mcr-4*, *mcr-5*), high-level tigecycline [MIC ≥ 4 µg/mL; *tet*(X3), *tet*(X4), *tet*(X5), *tet*(X6)], and vancomycin (*vanABCDEGHMNRSTUVXYZ*) [Figure 3B]. Among these, *vanR* was detected at the highest abundance, followed by *vanS*, *vanH*, *vanT*, and *vanY* [Figure 3C and Supplementary Figure 2]. The abundance of these genes in the upper respiratory tract of cats was higher in South China (including Southwest and Southern China) compared to other regions [Figure 3D]. Notably, a statistically significant difference in gene abundance was observed between cats in catteries and those in stray bases [Figure 3E].

### Distribution of main bacterial species associated with AMR that cause global burdens

**Figure 4:**
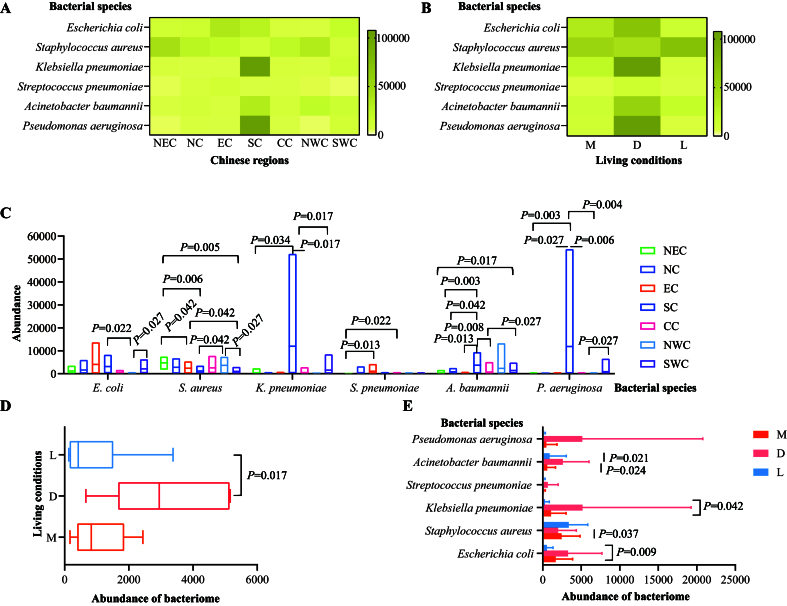
Characterization of the six primary bacterial pathogens associated with deaths due to AMR worldwide. (A) A heatmap illustrating the distribution and abundance of these six bacterial species in the upper respiratory tract of cats across various Chinese regions; (B) A heatmap displaying the distribution and abundance of these six bacterial species in the upper respiratory tract of cats in different living conditions; (C) Comparisons of the abundances of the bacteriome of these six bacterial species in the upper respiratory tract of cats from different Chinese regions; (D) Comparisons of the total abundances of the bacteriome of these six bacterial species in the upper respiratory tract of cats in different living conditions; (E) Comparisons of the abundances of the bacteriome of each of these six bacterial species in the upper respiratory tract of cats across different living conditions. Mann-Whitney U test was performed to compare the abundances between two groups, using pairwise multiple comparison adjustments according to the Benjamini-Hochberg procedure. The significance level was set at a *P* value of < 0.05 (*) and *P* > 0.05 is not marked. Different Chinese regions: NEC - Northeast China, NC - Northern China, EC - Eastern China, CC - Central China, SC - Southern China, NWC - Northwest China, SWC - Southwest China. Different living conditions: M - catteries, D - animal hospitals, L - stray bases. AMR: Antimicrobial resistance.

Antimicrobial-resistant bacteria were identified through metagenomic sequencing-based bacteriome analysis, revealing a total of 8,190 known bacterial species belonging to families such as *Enterobacteriaceae*, *Pasteurellaceae*, *Moraxellaceae*, and *Comamonadaceae* [Supplementary Figure 3 and Supplementary Table 4]. Notably, the six major pathogens linked to global AMR-related fatalities - *E. coli*, *S. aureus*, *K. pneumoniae*, *S. pneumoniae*, *A. baumannii*, and *P. aeruginosa*^[3]^ - were widely detected in cats across different regions and living conditions [Figure 4A and B]. While no significant difference was observed in the overall abundance of these bacteria among regions (*P* > 0.05), variations in the abundances of individual species were noted [Figure 4C]. Additionally, the combined abundance of these six pathogens in the upper respiratory tract of cats from animal hospitals was significantly higher than in those from stray bases [Figure 4D]. Specifically, *A. baumannii*, *K. pneumoniae*, *S. aureus*, and *E. coli* exhibited statistically significant differences in abundance depending on living conditions [Figure 4E].

**Figure 5:**
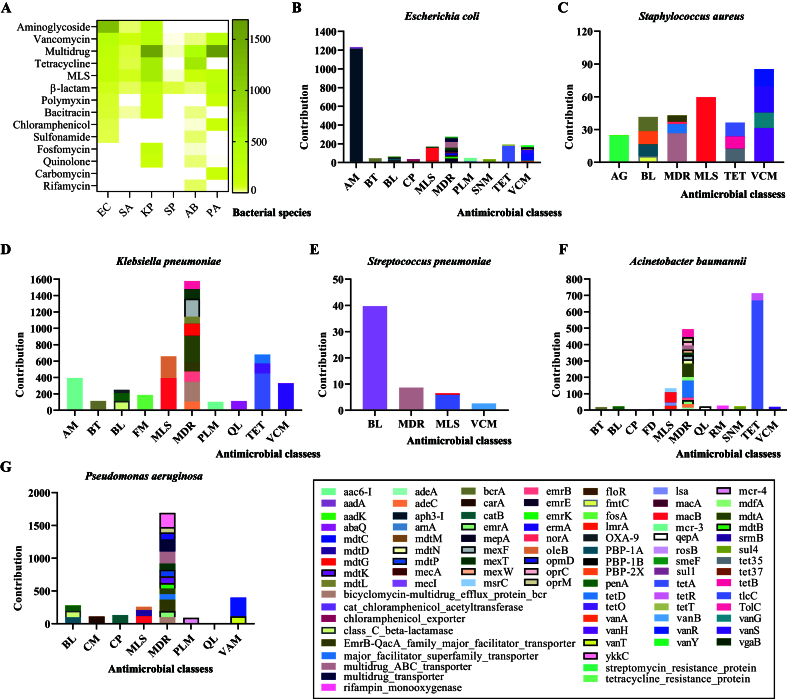
Contributions of the six primary bacterial pathogens to the abundance of genes mediating resistance against various antimicrobial classes. (A) A heatmap illustrating the contributions of the six leading bacterial pathogens to the resistant phenotypes against different antimicrobial classes; (B) A column chart showing the contributions of *Escherichia coli* to the abundance of genes mediating resistance against various antimicrobial classes; (C) A column chart showing the contributions of *Staphylococcus aureus* to the abundance of genes mediating resistance against various antimicrobial classes; (D) A column chart showing the contributions of *Klebsiella pneumoniae* to the abundance of genes mediating resistance against various antimicrobial classes; (E) A column chart showing the contributions of *Streptococcus pneumoniae* to the abundance of genes mediating resistance against various antimicrobial classes; (F) A column chart showing the contributions of *Acinetobacter baumannii* to the abundance of genes mediating resistance against various antimicrobial classes; (G) A column chart showing the contributions of *Pseudomonas aeruginosa* to the abundance of genes mediating resistance against various antimicrobial classes. Bacterial species: EC - *E. coli*, SA - *S. aureus*, KP - *K. pneumoniae*, SP - *S. pneumoniae*, AB - *A. baumannii*, PA - *P. aeruginosa*.

Further analysis clarified the contributions of different bacterial species to AMR, revealing distinct associations with specific ARGs [Figure 5]. The six aforementioned pathogens played critical roles in conferring resistance to key antimicrobials, including those classified by the WHO as highest-priority critically important agents [Figure 5]. Among them, *E. coli* was primarily associated with resistance to aminoglycosides and tetracyclines, mainly linked to *aph3-I* and *tetA*, respectively [Figure 5A and B]. *S. aureus* was notably linked to vancomycin resistance, primarily associated with *vanS* [Figure 5A and C]. *K. pneumoniae* contributed to resistance against β-lactams, tetracyclines, and the macrolide-lincosamide-streptogramin (MLS) antimicrobials, with key associations to class C β-lactamase coding gene (β-lactams), *penA* (β-lactams), *bla*_OXA-9_ (β-lactams), *tetO* (tetracyclines), *tetD* (tetracyclines), *macB* (MLSs), and *oleB* (MLSs), respectively [Figure 5A and D]. *S. pneumoniae* was implicated in MLS resistance, primarily linked to *tlrC* [Figure 5A and E]. *A. baumannii* mainly contributed to tetracycline resistance, associated with *tetA* [Figure 5A and F]. *P. aeruginosa* was linked to β-lactam resistance, primarily through class C β-lactamase-encoding gene and *penA* [Figure 5A and G]. Notably, *P. aeruginosa* and *K. pneumoniae* were the primary contributors to the MDR phenotype, albeit through different mechanisms. In *P. aeruginosa*, resistance was mainly driven by multidrug transporters, particularly Resistance-Nodulation-Division (RND) multidrug efflux transporters, whereas in *K. pneumoniae*, resistance was primarily mediated by multidrug efflux pumps such as MdtA, MexF, and EmrB [Figure 5A, D, and G].

### Bacterial species with public health concerns characterized in the upper respiratory tract of cats

**Figure 6:**
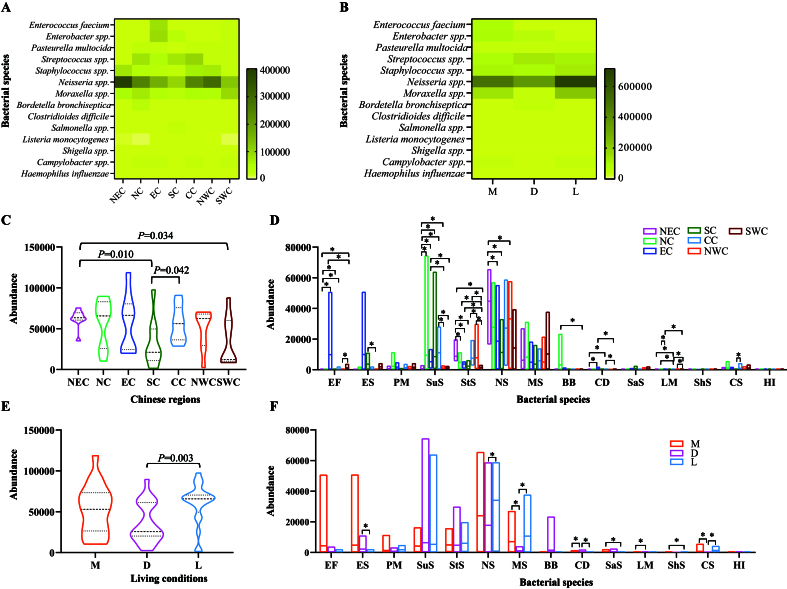
Characterization of bacterial species associated with public health concerns. (A) A heatmap displaying the distribution and detection abundance of specific bacterial species linked to public health concerns in the upper respiratory tract of cats from various Chinese regions; (B) A heatmap illustrating the distribution and detection abundance of specific bacterial species associated with public health concerns in the upper respiratory tract of cats across different living conditions; (C) Comparisons of the total abundances of the bacteriome of these bacterial species in the upper respiratory tract of cats from different Chinese regions (*P* < 0.05); (D) Comparisons of the abundances of bacteriome of different bacterial species in the upper respiratory tract of cats across various Chinese regions; (E) Comparisons of the total abundances of the bacteriome of these bacterial species in the upper respiratory tract of cats in different living conditions; (F) Comparisons of the abundances of the bacteriome of different bacterial species in the upper respiratory tract of cats across diverse living conditions. Mann-Whitney U test was performed to compare the abundances between two groups, using pairwise multiple comparison adjustments according to the Benjamini-Hochberg procedure. The significance level was set at a *P* value of < 0.05 (*) and *P* > 0.05 is not marked. Different Chinese regions: NEC - Northeast China, NC - Northern China, EC - Eastern China, CC - Central China, SC - Southern China, NWC - Northwest China, SWC - Southwest China. Different living conditions: M - catteries, D - animal hospitals, L - stray bases. Bacterial species: EF - *Enterococcus faecium*, ES - *Enterobacter* spp., PM - *Pasteurella multocida*, SuS - *Streptococcus* spp., StS - *Staphylococcus* spp., NS - *Neisseria* spp., MS - *Moraxella* spp., BB - *Bordetella bronchiseptica*, CD - *Clostridioides difficile*, SaS - *Salmonella* spp., LM - *Listeria monocytogenes*, ShS - *Shigella* spp., CS - *Campylobacter* spp., HI - *Haemophilus influenzae*.

Metagenomic sequencing revealed the presence of bacterial species with significant public health implications due to their association with human infections following cat exposure [Supplementary Figure 3]. Of particular concern were *Enterococcus faecium*, *Enterobacter* spp., *Pasteurella multocida*, *Streptococcus* spp., *Staphylococcus* spp., *Neisseria* spp., *Moraxella* spp., *Bordetella bronchiseptica*, *Clostridioides difficile*, *Salmonella* spp., *Listeria monocytogenes*, *Shigella* spp., *Campylobacter* spp., and *Haemophilus influenzae*. These pathogens were detected in the upper respiratory tract of cats across various Chinese regions [Figure 6A] and under different living conditions [Figure 6B], with many showing high detection abundances [Figure 6A and B]. Notably, the abundances of these pathogens were higher in cats from Northeast China compared to those from Southern China and Southwest China [Figure 6C]. Additionally, cats from Central China also harbored higher pathogen abundances than those from Southern China [Figure 6C]. Specifically, regional differences were observed in the abundances of most bacterial species, excluding *P. multocida*, *Moraxella* spp., *Salmonella* spp., *Shigella* spp., and *H. influenzae* [Figure 6D and Supplementary Table 5]. Interestingly, the total abundance of these pathogens in the upper respiratory tract of cats from animal hospitals was significantly lower than in those from stray bases [Figure 6E]. This difference was primarily driven by higher abundances of *P. multocida*, *Staphylococcus* spp., *Neisseria* spp., *Moraxella* spp., *C. difficile*, *Campylobacter* spp., and *H. influenzae* in stray cats [Figure 6F and Supplementary Table 6].

## DISCUSSION

In this study, we conducted a nationwide genomic survey to characterize the resistome and bacteriome in the upper respiratory tract of domestic cats in China. As one of the world’s fastest-growing economies, China has experienced a dramatic increase in pet ownership, particularly cats, in recent years. By 2022, an estimated 65.4 million cats were kept as pets in urban households in China^[33]^. Given this close human-animal interaction, understanding the antimicrobial-resistant bacterial profile in domestic cats carries significant public health implications. While several epidemiological studies have monitored the prevalence of zoonotic antimicrobial-resistant bacteria in Chinese cats^[34,35]^, many remain limited in scope. To our knowledge, this study represents the first large-scale metagenomic sequencing-based surveillance of antimicrobial-resistome and bacteriome in the upper respiratory tract of domestic cats across China.

By analyzing 1,454 samples, we comprehensively characterized AMR bacteria in feline respiratory tracts. Compared to other anatomical sites, the upper respiratory tract poses a higher transmission risk to humans due to frequent close contact. Notably, respiratory infections caused by antimicrobial-resistant bacteria contribute substantially to global AMR-related deaths^[3]^. Additionally, increasing reports of human infections following cat bites or scratches further justify our focus on oropharyngeal/nasal swabs^[36-38]^. These factors collectively highlight the importance of this study.

Our results indicate that domestic cats may serve as reservoirs for ARGs, which were abundantly detected in their upper respiratory tracts. Many of these ARGs confer resistance to antibiotics critical for human medicine, as classified by the WHO^[32]^, including: 3rd/4th/5th-generation cephalosporins (*bla*_CTX-M_, *bla*_TEM-1_, *bla*_SHV-1_, *bla*_VEB-5_, *bla*_GES-13_, *bla*_OXA-9/59/209_), vancomycin (*vanABCDEGHMNRSTUVXYZ*), polymyxins (*mcr-1.2*, *mcr-1.4*, *mcr-1.5*, *mcr-2.1*, *mcr-2.2*, *mcr-3*, *mcr-4*, *mcr-5*), and quinolones (*qnrA*, *qnrB*, *qnrS*). Of particular concern were mobile ARGs conferring resistance to last-resort antibiotics such as carbapenems (*bla*_NDM_, *bla*_VIM_, *bla*_OXA-244_), colistin (*mcr*), and high-level tigecycline [*tet*(X3), *tet*(X4), *tet*(X5), *tet*(X6)]. These antibiotics are vital for treating multidrug-resistant Gram-negative infections in humans, yet many are not approved for veterinary use in China^[39]^. The proximity of domestic cats to humans may explain their carriage of human-specific ARGs, but it also raises concerns about potential zoonotic transmission. To advance the One Health approach, proactive measures are urgently needed to mitigate AMR in cats and other animals^[40,41]^.

Beyond ARGs, we identified numerous bacterial species capable of causing severe infections in humans, including ESKAPE pathogens (*E. faecium*, *Enterobacter* spp., *S. aureus*, *K. pneumoniae*, *A. baumannii*, and *P. aeruginosa*), which pose major challenges in global healthcare^[42,43]^. Among them, *E. coli*, *S. aureus*, *K. pneumoniae*, *S. pneumoniae*, *A. baumannii*, and *P. aeruginosa* have been identified as the six primary pathogens linked to deaths attributed to AMR^[3]^. Additionally, *P. multocida*, *Streptococcus* spp., *Staphylococcus* spp., *Neisseria* spp., *Moraxella* spp., and *B. bronchiseptica* are known to cause infections in humans after cat bites or scratches^[44-46]^. For the other bacterial species, *C. difficile*, *Salmonella* spp., *L. monocytogenes*, *Shigella* spp., *Campylobacter* spp., and *H. influenzae* have been flagged by WHO and/or the United States Centers for Disease Control and Prevention (CDC) as significant public health concerns^[47]^. Many of these bacteria are part of the normal oral microbiota of healthy cats but can be transmitted to humans through direct contact, ectoparasites, or aerosols^[10,11]^.

Several identified bacteria are also causative agents of respiratory disease in cats. For example, *B. bronchiseptica* is a leading cause of feline respiratory infections^[48]^, while *Streptococcus* spp., *P. multocida*, *K. pneumoniae*, and *E. coli* often act as secondary invaders in immunocompromised or virally infected cats^[49]^. While antimicrobial therapy remains the primary treatment, drug selection should follow guidelines due to intrinsic resistance^[48]^. For example, *B. bronchiseptica* typically resists β-lactams due to the presence of species-specific β-lactamase encoded by *bla*_BOR-1_, rendering this class ineffective despite its common use for treating respiratory infections^[48,50]^.

This study has several limitations that should be acknowledged. To improve dataset comprehensiveness, future research should include samples from a wider variety of cat breeds and geographic regions. Additionally, while stray base cats were included, many had been in captivity, potentially limiting their representativeness of truly feral populations. However, sampling fully free-roaming cats is logistically challenging due to their elusive behavior. Due to the high cost of individual sequencing, pooled samples were used - a common and acceptable approach for resistome, bacteriome, and virome studies^[30,51,52]^, despite potential limitations in tracking ARGs and bacterial species at the individual level. Furthermore, antibiotic usage data were unavailable, which may influence resistome differences. Future work should incorporate detailed drug administration records to distinguish environmental effects from antibiotic-driven selection.

Despite these limitations, our findings still provide valuable insights into the AMR landscape of domestic cats across China and highlight their potential zoonotic risks. Regular health monitoring of cats under the One Health framework is essential to address global public health challenges.

## DECLARATIONS

### Availability of data and materials

All sequencing data were deposited into the NCBI Sequence Read Archive (SRA) database under the BioProject accession number PRJNA998709. SRA accession numbers are SRR33674915-SRR33674924, SRR33696099-SRR33696115, and SRR34261881-SRR34261890.

### Copyright

© The Author(s) 2025.
