# Supplementary material for: Profiling the composition of resistome and bacteriome in the upper respiratory tract of domestic cats with respiratory signs in China
Source: Microbiome Res Rep. 2025 Jul 28;4(3):27. doi: 10.20517/mrr.2025.04 (PMC12540054; doi:10.20517/mrr.2025.04)
Supplement: Supplementary file 1 [file mrr-4-3-27-SupplementaryMaterials.zip › mrr4004-Supplementary Figures.pdf]

Supplementary Materials

Qiuyan Li[1,2,3,#], Dengyuan Zhou[1,2,3,#], Longlong Cao[1,2,3], Yongfan Li[1,2,3], Jiakang Li[1,2,3], Jing Ye[1,2,3], Huanchun Chen[1,2,3], Jiangchao Zhao[4], Shengbo Cao[1,2,3], Zhong Peng[1,2,3]

[1]National Key Laboratory of Agricultural Microbiology, College of Veterinary Medicine, Huazhong Agricultural University, Wuhan 430070, Hubei, China.

[2]Hubei Hongshan Laboratory, Wuhan 430070, Hubei, China.

[3]Frontiers Science Center for Animal Breeding and Sustainable Production, The Cooperative Innovation Center for Sustainable Pig Production, Huazhong Agricultural University, Wuhan 430070, Hubei, China.

[4]Department of Animal Science, Division of Agriculture, University of Arkansas, Fayetteville, AR 72701, USA.

Correspondence to: Prof. Shengbo Cao, Prof. Zhong Peng, National Key Laboratory of Agricultural Microbiology, College of Veterinary Medicine, Huazhong Agricultural University, NO.1 Shizishan Street, Wuhan 430070, Hubei, China. E-mail: sbcao@mail.hzau.edu.cn; pengzhong@mail.hzau.edu.cn

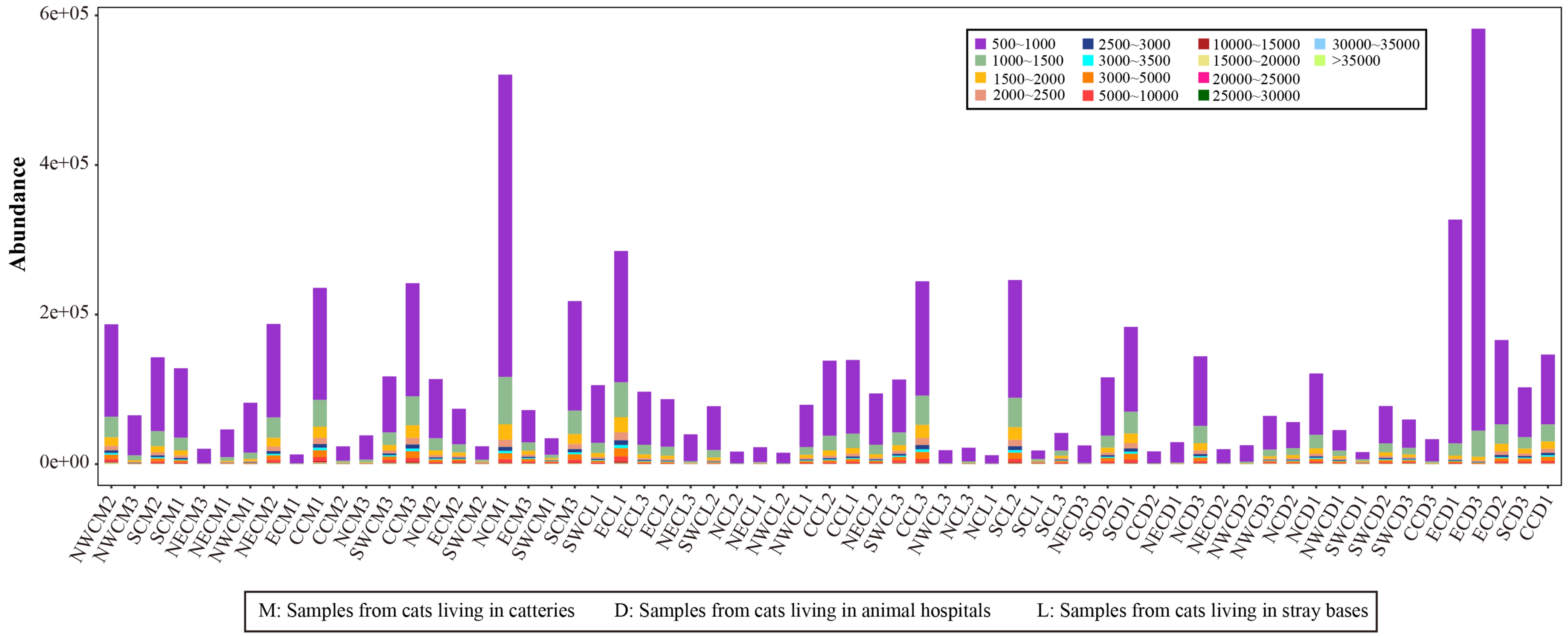

Supplementary Figure 1. A column chart illustrating the distribution of contigs generated from metagenomic sequencing for further analysis. Different Chinese regions: NEC - Northeast China, NC - Northern China, EC - Eastern China, CC - Central China, SC - Southern China, NWC - Northwest China, SWC - Southwest China. Different living conditions: M - catteries, D - animal hospitals, L - stray bases.

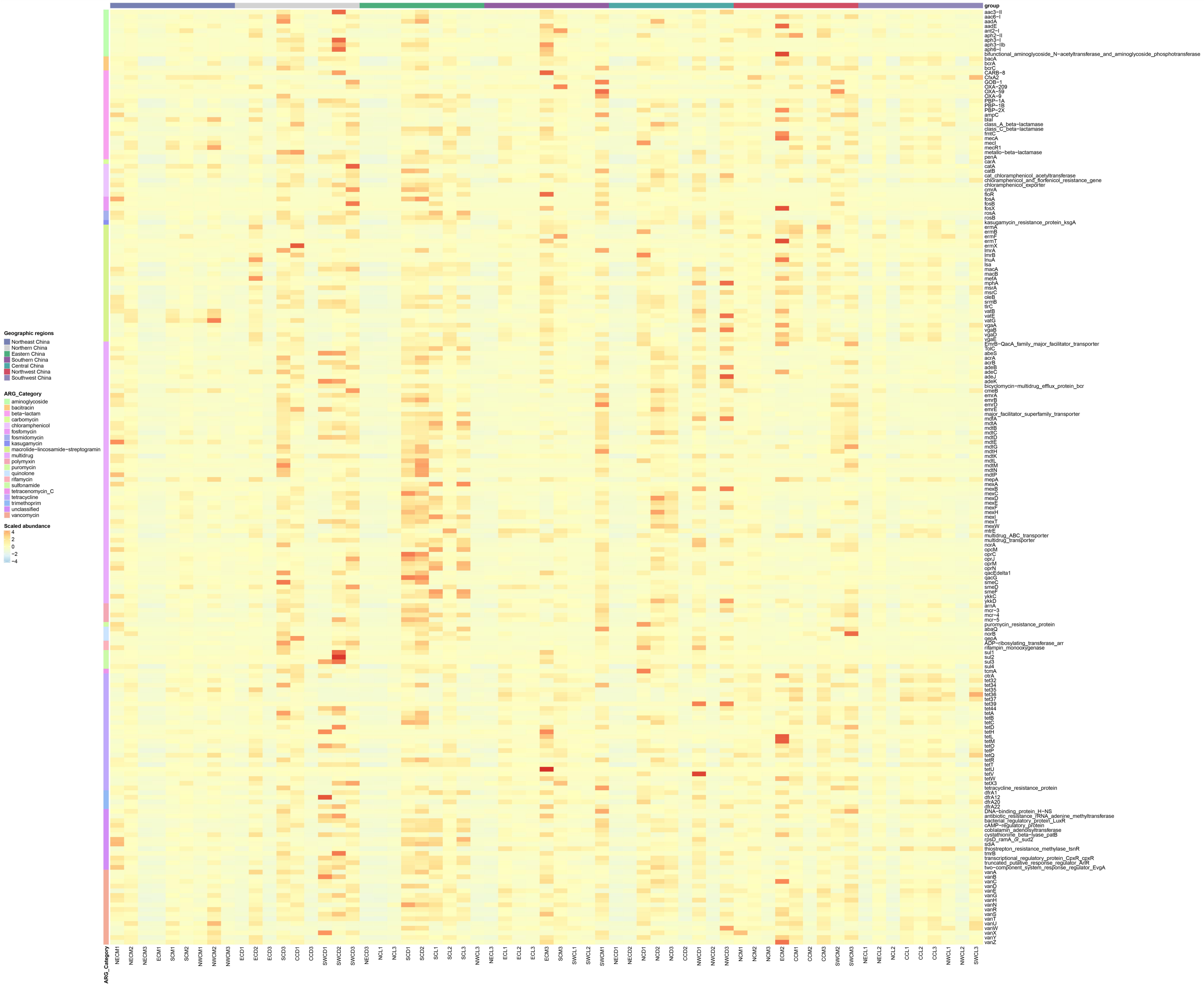
group
Geographic regions
Northeast China
Northern China
Eastern China
Southern China
Central China
Northwest China
Southwest China
ARG_Category
aminoglycoside
bacitracin
beta−lactam
carbomycin
chloramphenicol
fosfomycin
fosmidomycin
kasugamycin
macrolide−lincosamide−streptogramin
multidrug
polymyxin
puromycin
quinolone
rifamycin
sulfonamide
tetracenomycin_C
tetracycline
trimethoprim
unclassified
vancomycin
Scaled abundance
4
2
0
−2
−4
NECM1 NECM2 NECM3 ECM1 SCM1 SCM2 NWCM1 NWCM2 NWCM3 ECD1 ECD2 ECD3 SCD3 CCD1 CCD3 SWCD1 SWCD2 SWCD3 NECD3 NCL1 NCL3 SCD1 SCD2 SCL1 SCL2 SCL3 NWCL3 NECL3 ECL1 ECL2 ECL3 ECM3 SCM3 SWCL1 SWCL2 SWCM1 NECD1 NECD2 NCD1 NCD2 NCD3 CCD2 NWCD1 NWCD2 NWCD3 NCM1 NCM2 NCM3 ECM2 CCM1 CCM2 CCM3 SWCM2 SWCM3 NECL1 NECL2 NCL2 CCL1 CCL2 CCL3 NWCL1 NWCL2 SWCL3

Supplementary Figure 2. A heatmap showing the distribution of 444 antimicrobial resistance-associated genes in the upper respiratory tract of cats in different living conditions in seven Chinese regions. Different Chinese regions: NEC - Northeast China, NC - Northern China, EC - Eastern China, CC - Central China, SC - Southern China, NWC - Northwest China, SWC - Southwest China. Different living conditions: M - catteries, D - animal hospitals, L - stray bases.

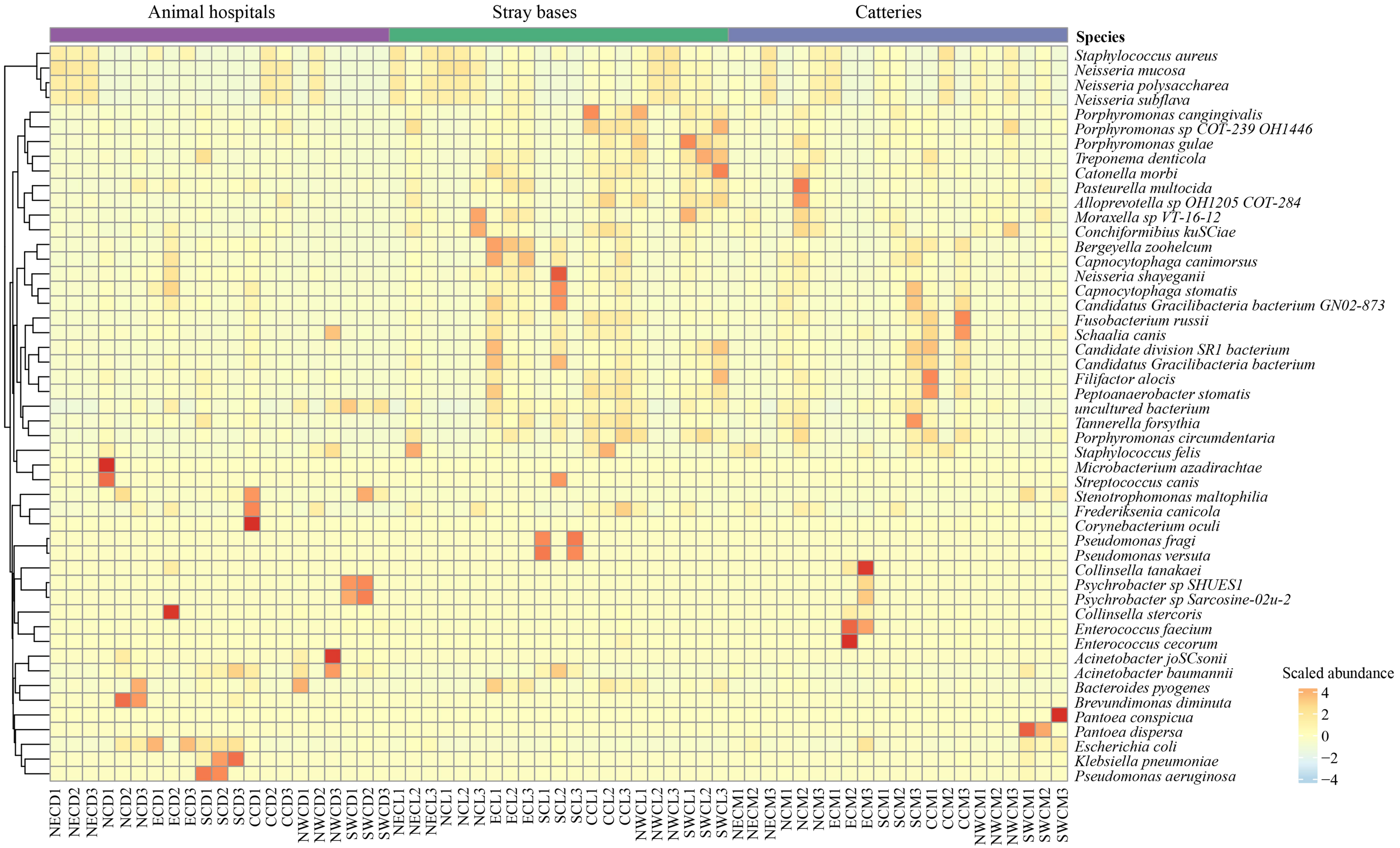

Animal hospitals
Stray bases
Catteries
Species
Staphylococcus aureus
Neisseria mucosa
Neisseria polysaccharea
Neisseria subflava
Porphyromonas cangingivalis
Porphyromonas sp COT-239 OH1446
Porphyromonas gulae
Treponema denticola
Catonella morbi
Pasteurella multocida
Alloprevotella sp OH1205 COT-284
Moraxella sp VT-16-12
Conchiformibius kuSCiae
Bergeyella zoohelcum
Capnocytophaga canimorsus
Neisseria shayeganii
Capnocytophaga stomatis
Candidatus Gracilibacteria bacterium GN02-873
Fusobacterium russii
Schaalia canis
Candidate division SR1 bacterium
Candidatus Gracilibacteria bacterium
Filifactor alocis
Peptoanaerobacter stomatis
uncultured bacterium
Tannerella forsythia
Porphyromonas circumdentaria
Staphylococcus felis
Microbacterium azadirachtae
Streptococcus canis
Stenotrophomonas maltophilia
Frederiksenia canicola
Corynebacterium oculi
Pseudomonas fragi
Pseudomonas versuta
Collinsella tanakaei
Psychrobacter sp SHUES1
Psychrobacter sp Sarcosine-02u-2
Collinsella stercoris
Enterococcus faecium
Enterococcus cecorum
Acinetobacter joSCsonii
Acinetobacter baumannii
Bacteroides pyogenes
Brevundimonas diminuta
Pantoea conspicua
Pantoea dispersa
Escherichia coli
Klebsiella pneumoniae
Pseudomonas aeruginosa
Scaled abundance
4
2
0
−2
−4
NECD1 NECD2 NECD3 NCD1 NCD2 NCD3 ECD1 ECD2 ECD3 SCD1 SCD2 SCD3 CCD1 CCD2 CCD3 NWCD1 NWCD2 NWCD3 SWCD1 SWCD2 SWCD3
NECL1 NECL2 NECL3 NCL1 NCL2 NCL3 ECL1 ECL2 ECL3 SCL1 SCL2 SCL3 CCL1 CCL2 CCL3 NWCL1 NWCL2 NWCL3 SWCL1 SWCL2 SWCL3
NECM1 NECM2 NECM3 NCM1 NCM2 NCM3 ECM1 ECM2 ECM3 SCM1 SCM2 SCM3 CCM1 CCM2 CCM3 NWCM1 NWCM2 NWCM3 SWCM1 SWCM2 SWCM3

Supplementary Figure 3. A heatmap showing the distribution of the top 50 zoonotic bacterial species in the upper respiratory tract of cats in different Chinese regions and/or living conditions. Different Chinese regions: NEC - Northeast China, NC - Northern China, EC - Eastern China, CC - Central China, SC - Southern China, NWC - Northwest China, SWC - Southwest China. Different living conditions: M - catteries, D - animal hospitals, L - stray bases.
